# Infective Larvae of *Brugia malayi* Induce Polarization of Host Macrophages that Helps in Immune Evasion

**DOI:** 10.3389/fimmu.2018.00194

**Published:** 2018-02-12

**Authors:** Aditi Sharma, Pankaj Sharma, Laxmi Ganga, Neha Satoeya, Shikha Mishra, Achchhe Lal Vishwakarma, Mrigank Srivastava

**Affiliations:** ^1^Parasitology Division, CSIR-Central Drug Research Institute, Lucknow, India; ^2^Academy of Scientific and Innovative Research (AcSIR), New Delhi, India; ^3^Sophisticated Analytical Instrument Facility (SAIF), CSIR-Central Drug Research Institute, Lucknow, India

**Keywords:** filariasis, polarization, M2 MΦ, regulatory MΦ, regulatory T-cell, regulatory B-cell, MAP kinase

## Abstract

Filarial parasites suppress, divert, or polarize the host immune response to aid their survival. However, mechanisms that govern the polarization of host MΦs during early filarial infection are not completely understood. In this study, we infected BALB/c mice with infective larvae stage-3 of *Brugia malayi* (Bm-L3) and studied their effect on the polarization of splenic MΦs. Results showed that MΦs displayed M2-phenotype by day 3 p.i. characterized by upregulated IL-4, but reduced IL-12 and Prostaglandin-D2 secretion. Increased arginase activity, higher arginase-1 but reduced NOS2 expression and poor phagocytic and antigen processing capacity was also observed. M2 MΦs supported T-cell proliferation and characteristically upregulated p-ERK but downregulated NF-κB-p65 and NF-κB-p50/105. Notably, Bm-L3 synergized with host regulatory T-cells (Tregs) and polarized M2 MΦs to regulatory MΦs (Mregs) by day 7 p.i., which secreted copious amounts of IL-10 and prostaglandin-E2. Mregs also showed upregulated expression levels of MHC-II, CD80, and CD86 and exhibited increased antigen-processing capacity but displayed impaired activation of NF-κB-p65 and NF-κB-p50/105. Neutralization of Tregs by anti-GITR + anti-CD25 antibodies checked the polarization of M2 MΦs to Mregs, decreased accumulation of regulatory B cells and inflammatory monocytes, and reduced secretion of IL-10, but enhanced IL-4 production and percentages of eosinophils, which led to Bm-L3 killing. In summary, we report hitherto undocumented effects of early Bm-L3 infection on the polarization of splenic MΦs and show how infective larvae deftly utilize the functional plasticity of host MΦs to establish themselves inside the host.

## Introduction

MΦs are sentinel immune cells that are centrally involved in antimicrobial defense, tissue repair, clearing cell debris, and maintaining homeostasis. Two major populations of MΦs have been identified and characterized in the mammalian system. M1 MΦs, also known as classically activated macrophages are induced by Th1 cytokine, IFN-γ, and exhibit a proinflammatory profile while M2 MΦs or alternatively activated MΦs are induced by Th2 cytokines and exhibit anti-inflammatory activities and tissue-repair functions (1, 2). In an *in vitro* setting, three different phenotypes of bone marrow-derived MΦ (BMMΦ) have been described based on whether these cells were primed with IFN-γ, LPS, immune complexes, or IL-4 (3). However, during *in vivo* conditions, MΦs being plastic in nature adapt to the surrounding stimuli and rapidly change their phenotype. In fact, it is this process of MΦ polarization that essentially regulates and decides the ultimate fate of the host immune response.

Filarial parasites stimulate the induction of M2 MΦs and impart profound functional changes in antigen-presenting cells viz. Dendritic cells (DCs) and MΦs that lead to an impaired Th1, but dominant Th2 immune response that provide protection during parasitic infections (4, 5). In addition to this, asymptomatic individuals harbor another phenotype of MΦs known as the regulatory MΦs (Mregs), which are characterized by high amounts of IL-10 that lead to modified type 2 responses and contribute to enhanced parasite survival. We also recently reported functional impairment of host DC subsets and attenuated T-cell response during early Bm-L3 infection (6). However, mechanisms that regulate the polarization of host MΦs following Bm-L3 infection remain unanswered. In the present study, we infected BALB/c mice with Bm-L3, and monitored the polarization of splenic MΦs during the first week of infection. We observed alternatively activated phenotype of splenic MΦs at day 3 p.i., which rapidly changed to a regulatory phenotype at day 7 p.i.; this shift was accompanied by accumulation of regulatory T cells (Tregs) in the spleens of infected mice and was guided by increased secretion of CC-chemokine 22 (CCL22) by splenic MΦs. Importantly, neutralization of Tregs activity by co-administration of anti-GITR + anti-CD25 function blocking antibodies checked the polarization of M2 MΦ to Mregs and resulted in reduced Bm-L3 burden. In conclusion, we show that Bm-L3 synergizes with host Tregs to subvert host immunity and establish itself during the first week of infection. Strategies that can prevent the polarization of host MΦs at the earliest host–parasite interface can help control or limit the progression of the disease.

## Materials and Methods

### Animals and Parasite

6–8 weeks old female BALB/c mice were used for all the experiments in accordance with our Institutional Animal Ethics Committee (IAEC) guidelines. Animals were housed in polypropylene cages (five animals per cage) and kept at our institute’s laboratory animal facility under standard pathogen-free conditions of temperature (24 ± 1°C) and humidity (55–68%) and fed standard pellet diet and water *ad libitum. Brugia malayi* was maintained in *Mastomys coucha* and the third infective larval stage of the parasite (Bm-L3, *n* = 50) recovered from infected *Aedes aegypti* were used to infect mice *via* the intra-peritoneal (i.p.) route. Control animals were administered sterile PBS (i.p.).

### Reagents

cDNA synthesis kit, SYBR green master mix, Trizol reagent, DQ-ovalbumin, anti-mouse monoclonal antibodies viz. F4/80, toll-like receptor (TLR)-2, TLR-4, TLR-6, TLR-9, CD69, FITC-labeled secondary IgG antibody, Annexin V Apoptosis Detection Kit, and Caspase sampler assay kit were purchased from Thermo Fischer Scientific (Waltham, MA, USA). Function blocking antibodies viz. anti-CD25 and anti-GITR were purchased from either Thermo Fischer Scientific or BioXcell (West Lebanon, USA). May Grunwald-Giemsa stain was purchased from Merck and Co. (Darmstadt, Germany). CD11c and CD4 magnetic cell separation kit (MACS) were purchased from Miltenyi Biotec (Bergisch-Gladbach, Germany). Fixation and permeabilization kit, Brefeldin A, cell strainer, RBC lysis buffer, anti-mouse monoclonal antibodies viz. CD11c, CD11b, CD80, CD86, MHC-II, TNF-α, IL-4, IL-12, IL-10, Gr-1, Siglec-F, CD4, CD25, FoxP3, CD19, CD5a, and CD1d were purchased from BD Biosciences (San Jose, CA, USA). Anti-mouse monoclonal antibodies Arginase-1 and NOS2 and CCL22 ELISA kit were purchased from R&D biosystems (Minneapolis, MN, USA). NF-κB, p-p38, and p-ERK antibodies were purchased from Cell Signaling Technology (Danvers, MA, USA). MEK inhibitor PD0325901, protein tyrosine phosphatase (PTP) inhibitor [bpv (phen)], FITC-dextran, and Arginase activity kit were purchased from Sigma (St. Louis, MO, USA). ELISA kits for Prostaglandins E2 (PGE2) and PGD2 were purchased from Cayman chemicals (Ann Arbor, MI, USA). ELISA kits for LXA4 and LXB4 were purchased from Elabscience (Bethesda, MD, USA). Tyrosine Phosphatase Assay kit was purchased from Promega (Madison, USA).

### Flow Cytometry

**Figure 1:**
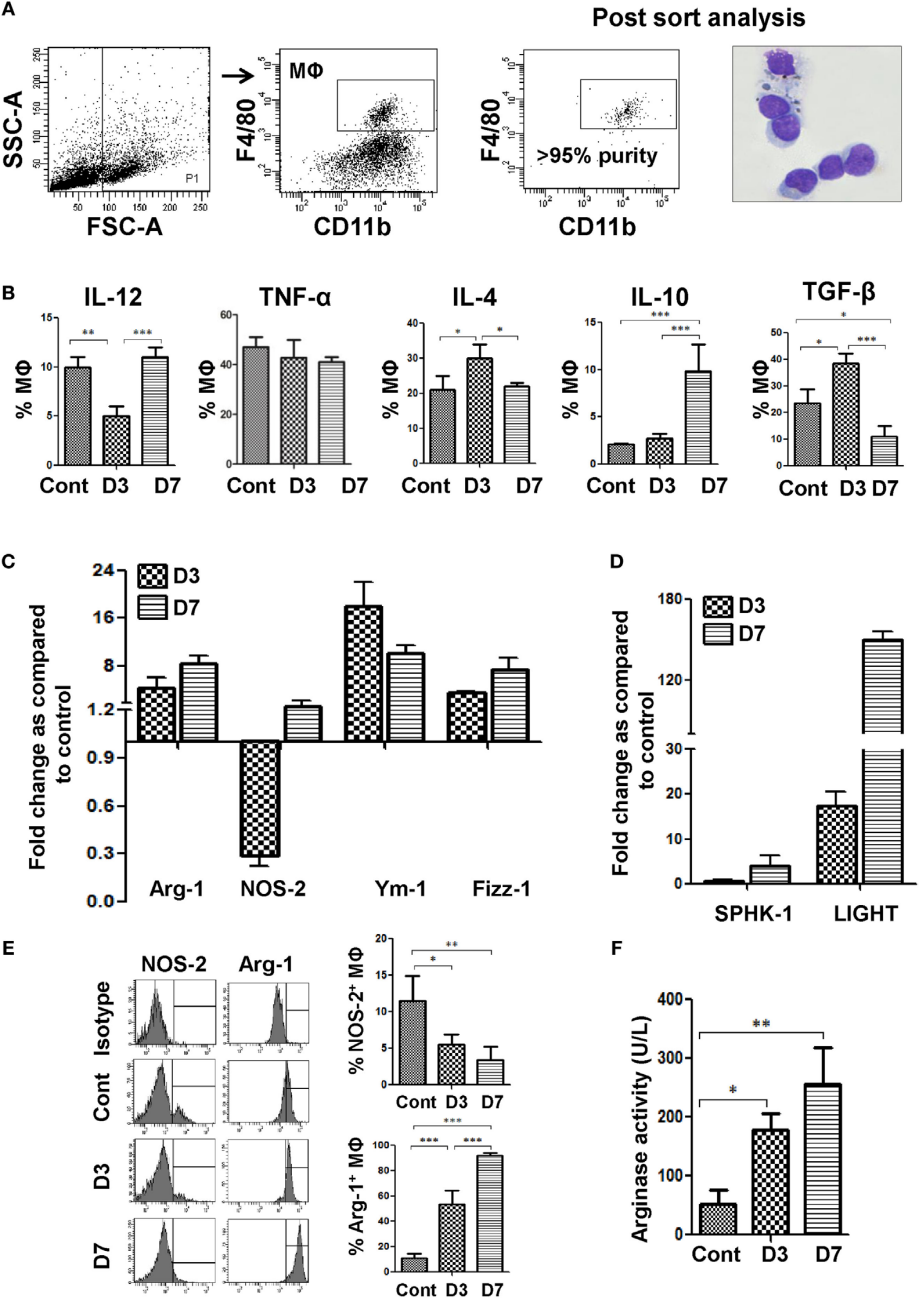
Bm-L3 infection causes polarization of host MΦs. **(A)** Gating strategy for the identification and sorting of splenic MΦs present in the CD11c positive cell fraction is shown. Post sort dot plot, and May-Grunwald–Giemsa-stained cytospins illustrate very high purity (≥95%) of sorted cells. **(B)** Percentages of splenic MΦs secreting IL-12, TNF-α, IL-4, IL-10, and TGF-β at indicated time points are shown. **(C,D)** Bar graphs represent fold change in the transcript levels of Arg-1, iNOS, Ym-1, and Fizz-1 **(C)** and Sphingosine kinase-1 (SPHK-1) and LIGHT **(D)** from FACS-sorted splenic MΦs at day 3 and day 7 post Bm-L3 infection as compared to control. The value set to 1 indicates no regulation, while values <1 indicate respective downregulation and values >1 indicate respective upregulation of a given gene at indicated time points post Bm-L3 infection. **(E)** Representative histograms show intracellular expression of Arg-1 and NOS-2 in splenic MΦs from uninfected control and Bm-L3-infected mice at day 3 and day 7 p.i. as determined by flow cytometry. Bar graphs on the right depict percentage (Mean ± SD) values of Arg-1^+^ and NOS-2^+^ splenic MΦs **(F)** Arginase activity in FACS-sorted splenic MΦs from uninfected control and Bm-L3 infected mice at day 3 and day 7 postinfection is shown. All values represent mean ± SD values from three independent experiments with at least 3–4 animals/group. *p*-Value of ≤0.05, ≤0.01, and ≤0.001 was considered significant, highly significant, and very highly significant and marked with *, **, and ***, respectively.

CD11c positive cells were enriched from the spleens of uninfected and Bm-L3 infected mice at day 3 and day 7 post infection using CD11c magnetic beads as described earlier (6). Thereafter, CD11c positive cell fraction (containing mostly DCs and MΦs) was incubated with CD11b-PE-Cy7 and F4/80-Pacific Blue anti-mouse monoclonal antibodies for 20 min at 4°C and flow cytometric data were acquired on five-decade log-scale dot plots displaying forward scatter (FSC) area vs. side scatter area (SSC). First hierarchy gate was set in FSC-A vs. SSC-A dot plot to exclude contaminating dead cells and cell debris, second hierarchy gate was set in FSC-A vs. FSC-H dot plot to exclude cell doublets and, thereafter, splenic MΦs present within the FSC-A vs. FSC-H dot plot were identified as CD11b^pos^, F4/80^pos^ cells as outlined in Figure 1A. Similarly, eosinophils, inflammatory monocytes, and regulatory B-cells (Bregs) were immunophenotyped from erythrocyte free single cell suspension of spleens based on the differential expression of following markers viz. Siglec F, MHC II, Gr-1, CD11b, CD19, CD1d, and CD5. Briefly, dot plot of FSC-A vs. SSC-A was used to identify granulocytes (low FSC-A, high SSC-A), and further sub-gating of this population helped in the identification of eosinophils (Siglec F^hi^, MHC II^neg^). Monocytes were identified as Gr-1^hi^, CD11b^pos^ cells, while Bregs were identified as CD19^+^, CD1d^mid^, CD5a^hi^ cells. Data were acquired on FACS Aria flow cytometer and compensation and data analysis was done using FACS DiVA software (BD Biosciences).

### Flow Cytometry Assisted Sorting of Splenic MΦs

Splenic MΦs were identified using the gating strategy as described above. MΦs were sorted using a high-speed FACS Aria flow cytometer fitted with a 70 µm nozzle as described recently (6–8). Sorted MΦs were subjected to post-sort analysis to ascertain the purity of sorted cells and a small fraction was used to prepare cytospins. Remaining cells were used for other immunological assays as detailed below.

### Estimation of Toll-Like Receptors (TLRs) and Intracellular Cytokines

CD11c^pos^ cells (*n* = 4 × 10^6^) were stained with CD11b-PE-Cy7 and F4/80-Pacific Blue monoclonal antibodies and distributed into four separate tubes followed by addition of FITC-labeled anti-mouse TLR-2, TLR-4, TLR-6, or TLR-9 antibodies. TLR expression on splenic MΦs was ascertained using the gating strategy as described above (Figure 1A). For estimating intracellular cytokines, CD11c^pos^ cells were first incubated with Brefeldin A (10 µg/ml) for 6 h, then washed and stained with CD11b-PE-Cy7 and F4/80-Pacific blue monoclonal antibodies. Next, they were fixed, permeabilized, and stained with PE-labeled anti-mouse TNF-α, TGF-β, IL-12, IL-4, and IL-10 monoclonal antibodies in separate tubes. Data were acquired on FACS Aria flow cytometer and compensation and data analysis was done using FACS DiVA software (BD Biosciences) (6, 7, 9).

### Analysis of Co-Stimulatory Molecules, Phagocytosis, and Antigen-Processing Capacity of Splenic MΦs

FACS-sorted splenic MΦs (*n* = 50,000) were incubated with either FITC-dextran (1 mg/ml) to estimate phagocytosis capacity, or with DQ-ovalbumin (0.5 mg/ml) to measure antigen processing capacity for 1 h in a CO_2_ incubator at 37°C and increase in FITC fluorescence was measured as described earlier (7). For analysis of maturation and co-stimulation markers, FACS-sorted splenic MΦs were incubated with anti-mouse monoclonal antibodies viz. CD80, CD86, and MHC-II at 4°C for 20 min and, thereafter, acquired on FACS Aria flow cytometer.

### T-Cell Proliferation Capacity of Splenic MΦs

FACS-sorted splenic MΦs (*n* = 5,000) were cocultured with CD4^+^ T cells (*n* = 2 × 10^5^) that were purified from the spleens of naïve mice using CD4 magnetic beads (Miltenyi Biotec). After 48 h, mitochondrial activity as a measure of T cell proliferation was measured by XTT assay (6).

### ELISA

Lipid intermediates viz. PGE2, PGD2, Lipoxin A4 (LXA4), and B4 (LXB4) present in the culture supernatant of flow-sorted splenic MΦs from control and Bm-L3-infected mice were quantified by ELISA as per instructions of the respective manufacturer.

### Inhibitor Studies

Bm-L3-infected mice were administered either MEK inhibitor, PD0325901 (5 mg/kg, i.p.) or PTP inhibitor [bpv (phen)] (0.8 mM, i.p.) in separate experiments. In one set of experiments, Bm-L3-infected mice received PD0325901 at day 0 and were sacrificed at day 3 p.i., while in another set they received two doses of PD0325901 at day 0 and day 3 p.i. and were sacrificed at day 7 p.i. Similarly, for PTP inhibition studies, Bm-L3 infected mice received 0.8 mM of [bpv (phen)] daily between day 4 and day 6 p.i. and were sacrificed at day 7 p.i. Bm-L3-infected mice that did not receive any inhibitor served as control animals for inhibition studies.

### Real-time RT PCR

Total RNA was isolated, quantified, and reverse transcribed from FACS-sorted splenic MΦs and reactions were run on Step One plus thermal cycler (Applied Biosystems) using the SYBR green master mix as described earlier (10). β-actin was used as the reference gene and mean fold-changes were calculated according to the 2^−ΔΔCT^ method (11). After reactions were over, melting curve analysis was performed to confirm the specificity of amplicons. Primer sequences used for RT-PCR are listed in Table 1.

**Table 1:** Primer sequences used for real-time RT-PCR.

Gene	Forward primer (5′-3′)	Reverse primer (5′-3′)
Ptpr a	TGGTTCATTCTTGTCCTGTTTGG	CTGTAGTAGCATTGTTGGCACT
Ptpr b	TCAAGGCAGGACAGTACCC	TGTATTTCTCCCATTCGCCTAGA
Ptpr c	CAGAAACGCCTAAGCCTAGTTG	ATGCAGGATCAGGTTTAGATGC
Ptpr d	AAATGGACTGAATACCGGATCAC	GAGGACCACTTGGAACATCTTC
Ptpr e	GATGACTGCAAGCGATTCCGA	GTTCCTTGTATGTGTCCAGATGG
Ptpr f	CTGCTCTCGTGATGCTTGGTT	ATCCACGTAATTCGAGGCTTG
Ptpr g	AGTCAGTCCGAGGGACAATTC	GGTGGCGTAGTCAAGGAGC
Ptpr h	CTTCTGGGGGATATGGGGTC	TCCCACACAAAGATCCATTTTCA
Ptpr j	GACTCAGGCGCTTCAGAATGT	TTGTTCAAGGTCTCATTGGTTGT
Ptpr k	CTCTGGCTTCTGTACCCGTG	GGACCCACTCAAAGTCATCGTAT
Ptpr m	CCGGAGAAACATTTTCAGGTGG	AGGTCGGTTTGGTCAGGGT
Ptpr n	CTTGTGTATGCGCCATCATTCG	GGTCCTGGTACTCAAAAGTAGTG
Ptpr n2	GAGGATGGCTTGTGTGGATCA	CGGAACCTTTTGACATCTTCCAA
Ptpr o	GCACACTTTTAATTGGACTGCTC	TGCCAGCTCCACATTCCCTA
Ptpr r	GAACGTGGTTGTGGACCCTC	TGGAGTCCTATGGGCTTCATT
Ptpr s	GGTGAACAACATACCCCCGAC	TCCCACCTCTGTGTAAGCCA
Ptpr t	CCATGCAGAAAAGCACCTCAT	CATGCTGGGACCACTTTCCA
Ptpr u	GGCTCAGTATGACGACTTCCA	GAACTGCACACAATGGGTGTC
Ptpr z1	GGAGAAGAACAGAACATCGTCC	TCATTGCTCTGGTAATAGCCCA
Ptp n1	GGAACTGGGCGGCTATTTACC	CAAAAGGGCTGACATCTCGGT
Ptp n11	AGAGGGAAGAGCAAATGTGTCA	CTGTGTTTCCTTGTCCGACCT
Ptp n12	ATGGAGCAAGTGGAGATCCTG	TCTCAATCGCATGAAGTCCCG
Ptp n13	TGGGCTGTATTAAATCAGAGTGC	CGGTGCGGTGAATGCTCTAA
Ptp n14	ACAGAAGAGACCGCTGATGTT	CTCTGAGCCTCTCGTCTATGG
Ptp n2	GCAGTGAGAGCATTCTACGGA	TGACACAAACCCCATCTTAGTGA
Ptp n21	TTGAAATTGAAACGCACCCGA	GCCACAGCTTCTAGGCTCTC
Ptp n22	CAGCAACTACTGAAAGAAGCCC	AGGATAGATTTTGTCGGCCTTG
Ptp n23	ACAGAATGCTATTCGCGTTGC	CCTGCTCGTACTTGATGTCTTC
Ptp n3	TTACGTGCGTTGGGTGGAAG	AGCCATCTAAAAACCGTATGCTG
Ptp n4	TCCGCCTGGATAGACCACTT	GCCATATTGAACTGACTGGACTT
Ptp n5	TACCAAGTCTGCTGCTAGTCT	GCTCACTGATTGACGCCTGT
Ptp n6	GGACTTCTATGACCTGTACGGA	GCTGCGTGTAATACTCGACCA
Ptp n7	CTGCCGACCTTGTCTTTGG	AGCAGATGGGTTCAACCGTG
Ptp n9	TTCCACTGCTACAGAGAAACAAG	GTTGGGTCTCGAACATTCAAGAT
Ptp 4a1	CAATCCAACCAATGCGACCTT	GAGTAGTGTCGTAAGTTGCTTCA
Ptp 4a2	CCACCAATGCGACTCTCAACA	CCATCATCAAACGGCCAATCT
Ptp 4a3	CATCACTGTTGTGGACTGGC	TGGATGGCGTCCTCGTACTT
Ptp la	GCCAGCGACGAGAAGGAAG	CCGTCATGGCGATATTGTAGAA
Gapdh	AGGTCGGTGTGAACGGATTTG	TGTAGACCATGTAGTTGAGGTCA
Arg-1	CTCCAAGCCAAAGTCCTTAGAG	AGGAGCTGTCATTAGGGACATC
iNOS	GGAGTGACGGCAAACATGACT	TCGATGCACAACTGGGTGAAC
Ym-1	CAGGTCTGGCAATTCTTCTGAA	GTCTTGCTCATGTGTGTAAGTGA
Fizz-1	CCAATCCAGCTAACTATCCCTCC	ACCCAGTAGCAGTCATCCCA
Sphingosine kinase-1	TCCTGGAGGAGGCAGAGATA	GCTACACAGGGGTTTCTGGA
LIGHT	CTGCATCAACGTCTTGGAGA	GATACGTCAAGCCCCTCAAG

### Analysis of Intracellular Cell Signaling Molecules

FACS-sorted splenic MΦs (*n* = 200,000) were fixed, permeabilized, and stained with primary antibodies against p-p38 and p-ERK. Thereafter, FITC-labeled secondary IgG antibody was added, and after a brief incubation, cells were washed and acquired on a BD FACS Aria flow cytometer (12). For analysis of transcription factor NF-κB, flow-sorted splenic MΦs were lysed in RIPA buffer containing protease inhibitor cocktail and resolved on 10% SDS-PAGE followed by transfer onto nitrocellulose membrane. Next, membranes were blocked with 3% BSA and probed overnight with primary antibody followed by addition of HRP-conjugated secondary antibody and detected using ECL kit.

### Enzymatic Assays

FACS-sorted splenic MΦs (*n* = 2 × 10^6^) were lyzed in RIPA buffer containing protease inhibitor cocktail and centrifuged at 10,000×*g* for 15 min at 4°C. Protein tyrosine phosphatase *(*PTP*)* activity was determined in the supernatant according to instructions of the manufacturer (13). Arginase activity was similarly measured in the supernatant by comparing with a urea standard (14). For quantification of caspase activity, p-nitroaniline labeled peptide substrate was used and absorbance of free pNA was quantified using a spectrophotometer (15).

### Neutralization of Regulatory T Cells

To neutralize the activity of regulatory T cells, Bm-L3-infected mice were administered either respective IgG isotypes or combination of anti-CD25 + anti-GITR function blocking antibodies (100 µg each, i.v.) at day 4 and day 6 post Bm-L3 infection, 24 h later, spleens were excised and immunological assessments were carried out as described in the study.

### Apoptosis of Regulatory T Cells

Apoptosis of Tregs was assessed by Annexin V-FITC and propidium iodide (PI) staining pattern of CD4^+^CD25^+^ T cells (Tregs) present within the lymphocyte gate (low FSC-A, low SSC-A) as per the instructions of the manufacturer.

### Statistics

Figures represent mean ± SD values derived from at least three independent experiments having at least 3–4 animals/group. Statistical analysis was done by either one-way analysis of variance using Bonferroni’s test (Graph Pad Prism 5.0 version 5.01; Graph Pad, San Diego, CA, USA) or two tailed Student’s *t*-test. *p*-Value ≤0.05, ≤0.01, and ≤0.001 between different groups or time points were considered significant, highly significant and very highly significant and marked with *, ** and *** respectively.

## Results

### Infective Larvae of *Brugia malayi* (Bm-L3) Polarize Splenic MΦs

Splenic MΦs were immunophenotyped and sorted using flow-cytometry on day 3 and day 7 post Bm-L3 infection as described in the Section “Materials and Methods.” Post-sort analysis showed very high purity (>95%) of sorted cells (Figure 1A). Percentages of splenic MΦs-secreting IL-4 increased at day 3 p.i. (*p* ≤ 0.05), but those secreting IL-12 decreased (*p* ≤ 0.01) as compared to uninfected controls. This scenario changed rapidly at day 7 p.i. when splenic MΦs secreting IL-4 decreased significantly (*p* ≤ 0.05) while those secreting IL-12 and IL-10 increased as compared to day 3 p.i. (*p* ≤ 0.001 for both). Notably, percentages of splenic MΦs secreting TGF-β increased at day 3 p.i. (*p* ≤ 0.05) but dropped significantly at day 7 p.i. (*p* ≤ 0.001, Figure 1B).

Real-time RT-PCR analysis revealed 4.5-fold elevated mRNA expression of Arg-1 at day 3 p.i., which increased further to 8.5-fold at day 7 p.i. Quite contrary to this, mRNA expression of NOS2 in splenic MΦs decreased 3.5-fold at day 3 p.i., but moderately increased to 1.8-fold at day 7 p.i. when compared to uninfected controls (Figure 1C). Importantly, transcript levels of other alternative activation markers viz., Ym-1, which is associated with eosinophil chemotaxis increased 18-fold at day 3 p.i. and 10-fold at day 7 p.i. Similarly, Fizz-1, which dampens Th2 inflammation was upregulated 3.6- and 7.5-fold at day 3 and day 7 p.i., respectively (Figure 1C). Also, key markers asociated with regulatory macrophages, i.e., sphingosine kinase-1 and LIGHT were upregulated by 4- and 150-fold, respectively, at day 7 p.i. (Figure 1D), suggesting that Bm-L3-infected splenic MΦs were indeed phenotypically different at these two time points.

To further confirm our findings, we analyzed expression levels of Arg-1 and NOS2 *via* flow cytometry and the results showed 4.7- and 8-fold upregulated levels of Arg-1 in Bm-L3-infected splenic MΦs at day 3 and day 7 p.i., respectively, which was in contrast to 2.0- and 3.4-fold downregulated levels of NOS2 observed at day 3 and day 7 p.i., respectively (Figure 1E). Interestingly, this observation was corroborated by 3.5-fold (*p* ≤ 0.05) and five fold (*p* ≤ 0.01) increased arginase activity in Bm-L3-infected splenic MΦs at day 3 and day 7 p.i., respectively, as compared to uninfected control animals (Figure 1F). Taken together, these results reflected early perturbations in the cytokine secreting potential of splenic MΦs and underlined a tactical shift from a predominantly alternatively activated state (M2 phenotype) of splenic MΦs at day 3 p.i. to a more polarized regulatory phenotype (Mregs) at day 7 p.i. during the first week of Bm-L3 infection.

### Polarized Splenic MΦs Are Differentially Impaired during Bm-L3 Infection

**Figure 2:**
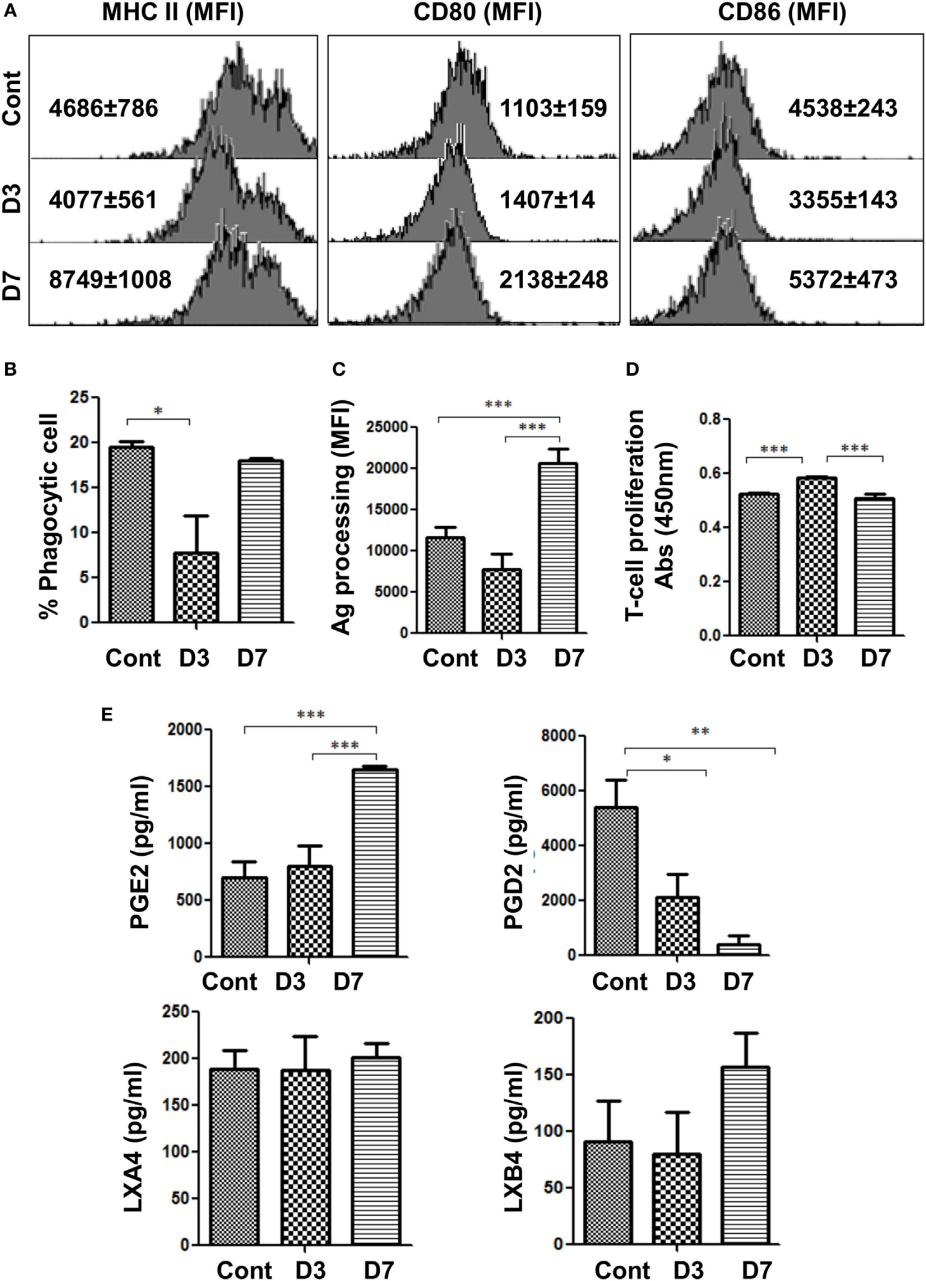
Bm-L3 infection differentially modulates the functions of host MΦs. **(A)** Histograms represent expression of maturation and costimulatory markers viz. MHC II, CD80, and CD86 on FACS-sorted splenic MΦs from uninfected control and Bm-L3 infected mice at day 3 and day 7 p.i. as determined by flow cytometry. Values in bracket are mean fluorescence intensity (MFI) values at given time points. **(B,C)** Antigen uptake (phagocytosis) and antigen-processing capacity of FACS-sorted splenic MΦs from uninfected control and Bm-L3-infected mice at day 3 and day 7 postinfection is shown. Values represent % phagocytic cell or MFI values as determined by FITC-Dextran uptake or DQ-ova-albumin cleavage, respectively. **(D)** Mitochondrial activity, as a measure of T cell proliferation was assessed by XTT assay. Figure represents OD values of FACS-sorted splenic MΦs cocultured with naïve CD4^+^ T cells at 450 nm at indicated time points. **(E)** Concentration of different lipid intermediates present in the culture supernatant of FACS-sorted splenic MΦs is shown. All values represent mean ± SD values from three independent experiments with at least 3–4 animals/group. *p*-value of ≤0.05, ≤0.01, and ≤0.001 was considered significant, highly significant, and very highly significant and marked with *, **, and ***, respectively.

We assessed the expression of maturation and co-stimulatory markers on the two phenotypes of splenic MΦs, i.e., M2 MΦs and Mregs at day 3 and day 7 p.i., respectively. While the expression of MHCII and CD86 decreased in M2 MΦs, that of CD80 did not change significantly at day 3 p.i. However, expression of these markers was elevated in Mregs at day 7 p.i. (Figure 2A). Furthermore, M2 MΦs exhibited decreased phagocytic capacity (Figure 2B) (*p* ≤ 0.05), which contrasted with significantly increased antigen processing capacity (*p* ≤ 0.001) observed in Mregs at day 7 p.i. (Figure 2C).

To further understand the functional consequences of these findings on the proliferation of T cells, FACS-sorted M2 MΦs (at day 3 p.i.) and Mregs (at day 7 p.i.) were cocultured with purified CD4^+^ T-cells obtained from naïve mice. Results showed that M2 MΦs supported the proliferation of CD4^+^ T cells (Figure 2D; *p* ≤ 0.001) Mregs did not, which underlined the suppressive nature of these cells. Moreover, significantly elevated level of Prostaglandin E2 (PGE2, *p* ≤ 0.001), but drastically decreased level of Prostaglandin D2 (PGD2, *p* ≤ 0.01) was observed in Mregs (Figure 2E), but quite surprisingly, no significant change was observed in the level of other small lipid intermediate viz. Lipoxin A4 (LXA4), and Lipoxin B4 (LXB4). These results showed that in addition to IL-10, elevated levels of PGE2 might have contributed toward polarization of M2 MΦs to Mregs during the early phase of Bm-L3 infection.

### MAP Kinases Regulate Polarization of Splenic MΦs

**Figure 3:**
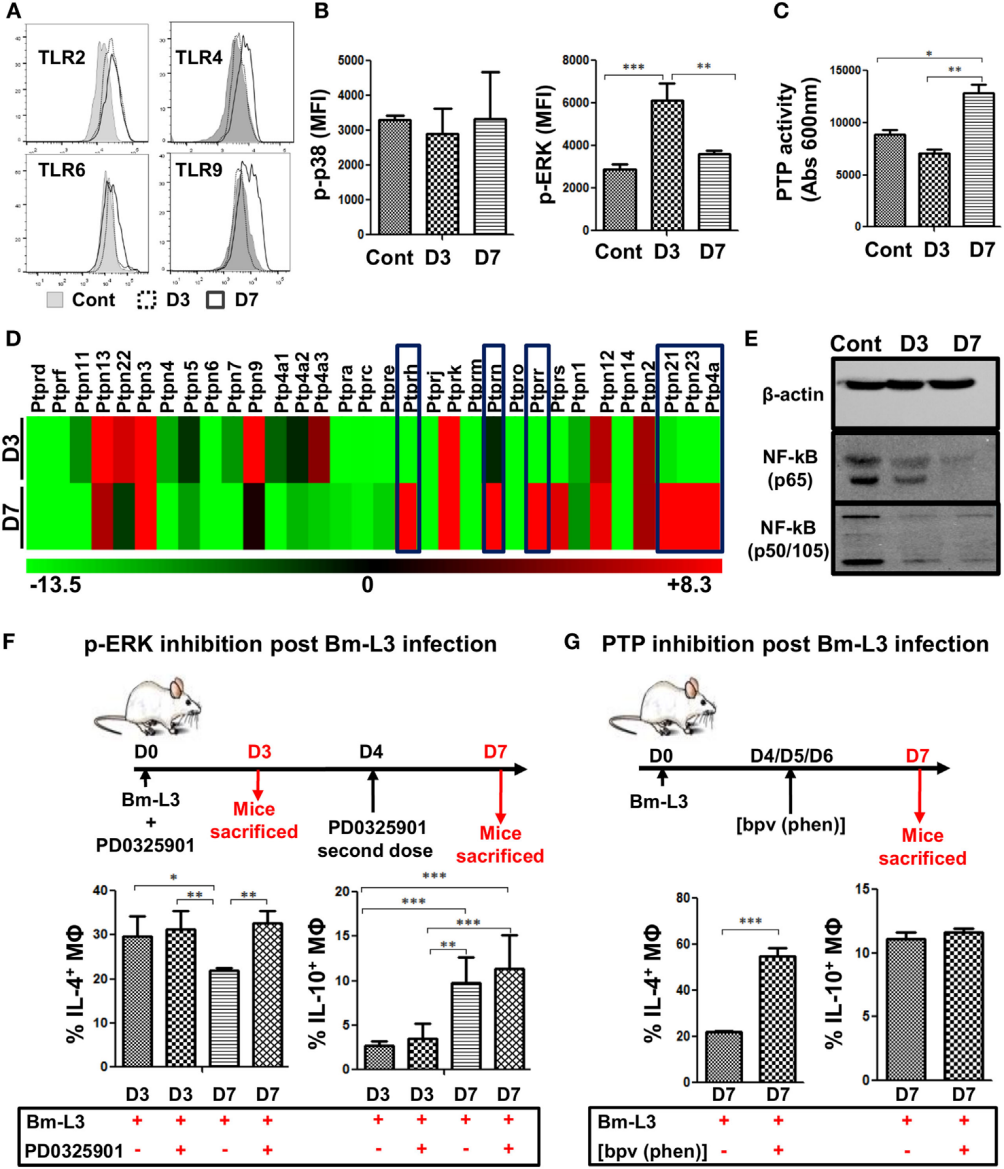
Bm-L3 infection impairs signaling in host MΦs. **(A)** Histograms represent expression of different toll like receptors (TLRs) on splenic MΦs from uninfected control and Bm-L3-infected mice at day 3 and day 7 p.i. **(B)** Phosphorylation of p38 and ERK in FACS-sorted splenic MΦs from uninfected control and Bm-L3 infected mice at day 3 and day 7 p.i. is shown. Values represent mean fluorescence intensity (MFI) values at given time points. **(C)** Bar graph represents protein tyrosine phosphatases (PTP) activity in FACS-sorted splenic MΦs in uninfected control and Bm-L3-infected mice at day 3 and day 7 p.i. **(D)** Heat map showing fold change (red-upregulated and green-downregulated of different PTPs in flow-sorted splenic MΦs at day 3 and day 7 post Bm-L3 infection as compared to uninfected control). **(E)** Representative western blot showing expression of β-actin, NFκB-p65, and NFκB-p50/105 in uninfected control and Bm-L3-infected mice at day 3 and day 7 p.i. **(F)** Schematic representation of the experimental strategy used for the inhibition of Erk. Bm-L3-infected mice were administered MEK inhibitor, PD0325901 at day 0 and day 4 p.i., and immunological assessments were made at day 3 and day 7 p.i., respectively. Figure depicts the percentages of IL-4 and IL-10 secreting MΦs at day 3 and day 7 before and after Erk inhibition. **(G)** Schematic representation of the experimental strategy used for the depletion of PTP activity. Bm-L3-infected mice were administered [bpv (phen)] daily from day 4 p.i. to day 6 p.i. and immunological assessments were made at day 7 p.i. Figure depicts the percentages of IL-4 and IL-10 secreting MΦs at day 7 before and after PTP inhibition. Values in **(B,C,F,G)** represent mean ± SD values from two independent experiments with at least 3–4 animals/group. *p*-Value of ≤0.05, ≤0.01, and ≤0.001 was considered significant, highly significant, and very highly significant and marked with *, **, and ***, respectively.

To gain deeper insights into the molecular pathways that regulated the polarization of splenic MΦs, we assessed the expression of toll-like receptors on MΦs *via* flow cytometry. Results presented as histograms (Figure 3A) along with mean fluorescence intensity (MFI) values given in Table 2 showed that expression of TLR2, TLR4, TLR6, and TLR9 increased in M2 MΦs and Mregs at day 3 at day 7 p.i. as compared to uninfected controls. However, no change was observed in the transcript level of TLR 5 between M2 MΦs and Mregs (data not shown). Furthermore, while the expression of p-p38 remained unaltered, that of p-ERK was significantly upregulated in M2 MΦs at day 3 p.i. (Figure 3B; *p* ≤ 0.001; MFI = 6,139 ± 785); however, it was downregulated in Mregs at day 7 p.i. (Figure 3B; *p* ≤ 0.01, MFI = 3,611 ± 164) when compared to uninfected controls (Figure 3B; MFI = 2,885 ± 243).

**Table 2:** Mean fluorescence intensity values of different toll-like receptors (TLRs) in splenic MΦs of control and Bm-L3 infected mice at day 3 and day 7 postinfection.

		TLR 2	TLR4	TLR6	TLR9
Splenic MΦs	Cont	13,606 ± 1,676	3,691 ± 141	28,233 ± 94	4,945 ± 308
Splenic MΦs (M2 MΦs)	D3	18,428 ± 1,624	4,144 ± 424	31,336 ± 4,850	5,863 ± 692
Splenic MΦs (Mregs)	D7	18,093 ± 1,736	4,384 ± 228	30,827 ± 2,481	6,095 ± 467

Additionally, since the activation of MAPKs is counter-regulated by several PTPs, we estimated PTP activity and found that it was significantly upregulated in Mregs at day 7 p.i. as compared to M2 MΦs (Figure 3C, *p* ≤ 0.01). Further mRNA analysis revealed that six different PTPs viz., Ptprh, Ptprn, Ptprr, Ptpn21, Ptpn23, and Ptp4a were significantly upregulated in Mregs, which suggested their involvement in inducing hypo-responsive state in Mregs (Figure 3D).

We also analyzed the phosphorylation pattern of two major subunits of NF-κB viz. p65 and p50/105 and found that expression of p65 and p50/105 was downregulated in M2 MΦs, but it was severely impaired in Mregs (Figure 3E). To confirm whether MAPKs regulated this process of polarization, we administered MEK inhibitor PD0325901 to Bm-L3-infected mice and found that ERK inhibition did not affect the development of M2 phenotype, but it significantly altered the polarization of M2 MΦs to Mregs as was evident from higher percentages of IL-4-secreting MΦs (*p* ≤ 0.05) at D7 post PD0325901 treatment (Figure 3F). Interestingly, administration of PD0325901 did not affect the IL-10 secreting potential of MΦs following treatment. Similarly, to confirm the role of PTPs in the polarization of M2 MΦs to Mregs, we administered PTP inhibitor [bpv (phen)] to Bm-L3-infected mice daily between day 4 and day 6 p.i. and found increased percentages of MΦs-secreting IL-4, but not IL-10 at day 7 p.i. (Figure 3G, *p* ≤ 0.001). These data suggest that complex mechanisms regulate the process of MΦ polarization and the parasite cleverly exploits these intricately wired molecular pathways to aid its survival.

### Infection with Bm-L3 Expands Regulatory T Cells in the Spleens of Mice

**Figure 4:**
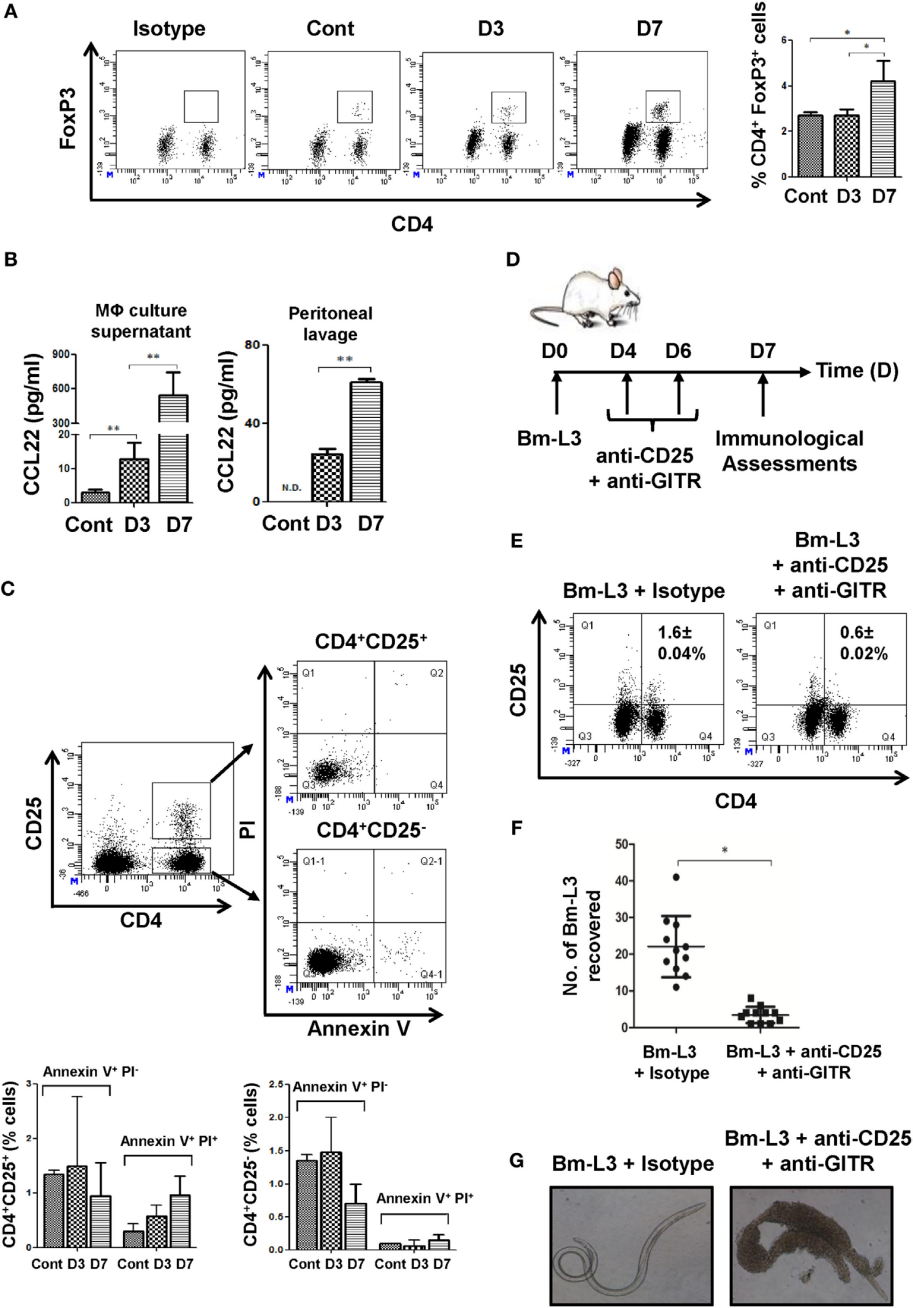
Neutralization of regulatory T-cells (Tregs) repolarizes splenic MΦs. **(A)** Representative flow cytometry dot plots showing CD4^+^Foxp3^+^ Tregs present in the spleens of uninfected control and Bm-L3 infected mice at day 3 and day 7 p.i. Bar graph on the right depict percentage (mean ± SD values) of CD4^+^Foxp3^+^ Tregs. **(B)** Concentration of CCL22 present in the culture supernatant of FACS-sorted splenic MΦs and peritoneal fluid of uninfected control and Bm-L3-infected mice at day 3 and day 7 p.i. **(C)** Representative flow cytometry dot plots showing apoptotic cells present within the CD4^+^ CD25^+^ and CD4^+^ CD25^−^ T cell gate. Histograms show the percentages of Annexin V^+^ PI^−^ and Annexin V^+^ PI^+^ cells present in control and Bm-L3-infected mice at day 3 and day 7 p.i., as enumerated from FACS dot plots **(D)** Schematic representation of experimental strategy used for the depletion of Tregs is shown. Bm-L3-infected mice were administered anti-CD25 + anti-GITR function blocking antibodies at day 4 and day 6 p.i., and immunological assessments were made at day 7 p.i. **(E)** Representative flow cytometry dot plots showing CD25^+^ CD4^+^ Tregs present in the spleens of anti-CD25 + anti-GITR treated mice or IgG administered controls at day 7 p.i. **(F)** Number of Bm-L3 recovered from the peritoneal cavity of anti-CD25 + anti-GITR-treated mice or IgG administered controls at day 7 p.i. **(G)** Representative bright field microscopy images showing adhesion of leukocytes (mainly eosinophils and MΦs) to the dead Bm-L3 recovered from the peritoneal cavity of anti-CD25 + anti-GITR treated mice or IgG administered controls at day 7 p.i. *p*-Value of ≤0.05, ≤0.01, and ≤0.001 was considered significant, highly significant, and very highly significant and marked with *, **, and ***, respectively.

Based on our previous observation of increased IL-10 secretion by Mregs at day 7 p.i., we reasoned if this would have a bearing on the numbers of Tregs present in the spleens of mice. Indeed, we observed increased percentages of Tregs at day 7 p.i. (4.2 ± 0.91%; Figure 4A) as compared to control animals (2.7 ± 0.14%, Figure 4A). This observation was also complemented by significantly increased concentration of CCL22, a Treg chemoattractant in the culture supernatant of FACS-sorted MΦs at day 7 p.i. (*p* ≤ 0.01, Figure 4B) and in the peritoneal lavage of Bm-L3-infected mice at day 7 p.i. (*p* ≤ 0.01, Figure 4B). Together, these results supported the notion that CCL22 driven Treg recruitment might have helped in the polarization M2 MΦs to Mregs. To further confirm our findings that Tregs were indeed driven by elevated CCL22 gradient and were not a result of *de novo* recruitment from the peripheral blood as might be the case during apoptosis, we checked the percentages of apoptotic T cells present within the CD4^+^CD25^+^ and CD4^+^CD25^−^ gate and quite expectedly found no difference between the control and Bm-L3-infected animals either at day 3 or day 7 p.i. (Figure 4C). To strengthen our reasoning even further, we blocked the activity of Tregs by co-administering neutralizing antibodies against CD25 and GITR (i.v.) at day 4 and day 6 post Bm-L3 infection (Figure 4D) and quite notably found significantly depleted percentages of Tregs (Figure 4E), along with drastically reduced Bm-L3 burden in treated animals (Figure 4F). Not only this, Bm-L3 recovered from anti-CD25 + anti-GITR treated animals were dead and surrounded by thousands of host cells attached to their surface, which showed that anti-CD25 + anti-GITR treatment suppressed the activity of Tregs and boosted anti-parasitic Th2 immunity (Figure 4G).

### Treatment with Anti-CD25 + Anti-GITR Antibodies Prevent Polarization of MΦs

**Figure 5:**
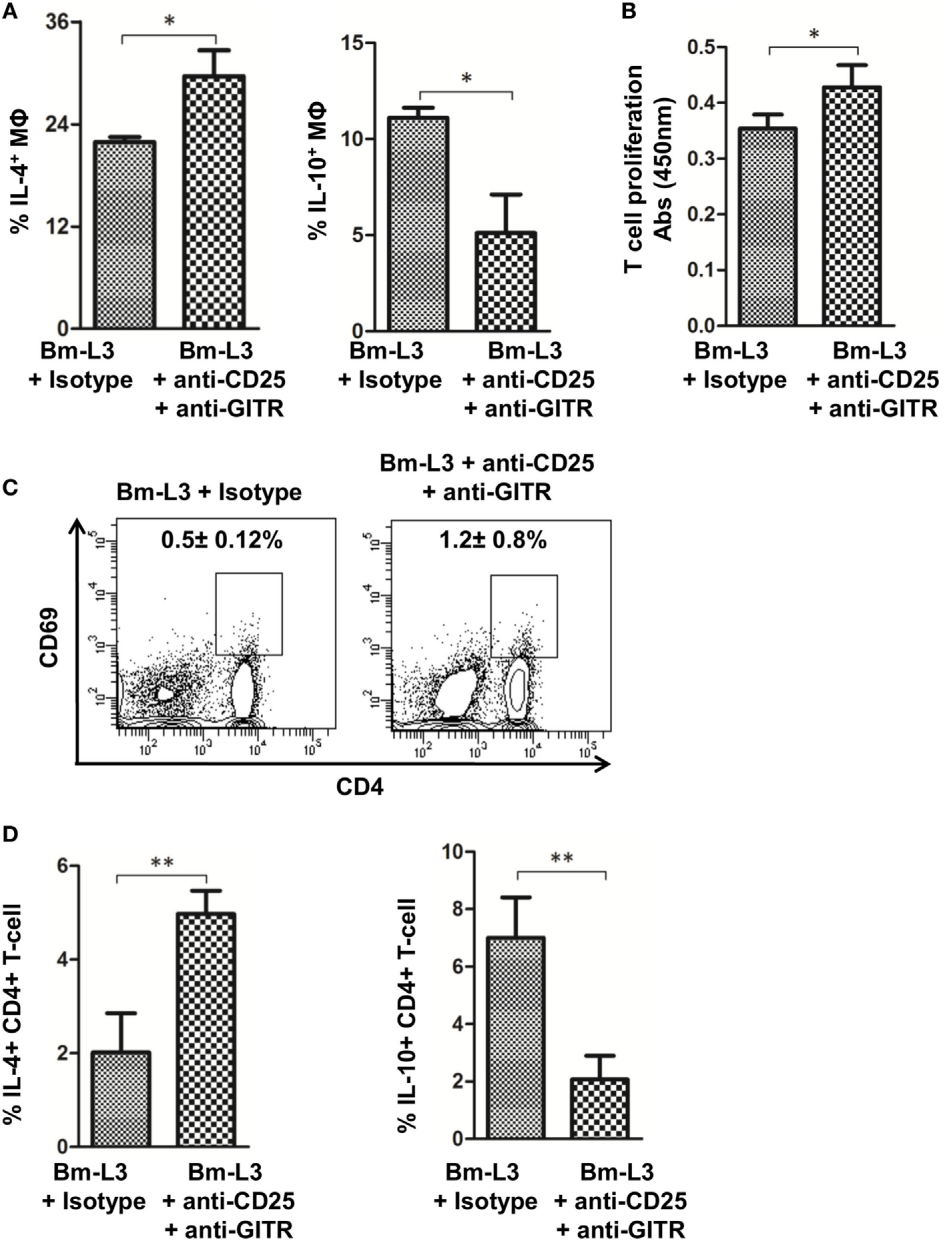
Neutralization of regulatory T-cell activity prevents polarization of splenic MΦs. **(A)** Bar graphs represent percentages of FACS-sorted splenic MΦs-secreting cytokines IL-10 and IL-4 from anti-CD25 + anti-GITR-treated mice or uninfected IgG control at day 7 p.i. **(B)** Mitochondrial activity, as a measure of T cell proliferation was assessed by XTT assay. Figure represents OD values of FACS-sorted splenic MΦs cocultured with naïve CD4^+^T cells at 450 nm at indicated time points from anti-CD25 + anti-GITR treated mice or uninfected IgG control at day 7 p.i. **(C)** Representative flow cytometry dot plots showing percentages of activated CD4^+^ T cells from anti-CD25 + anti-GITR treated mice or uninfected IgG control at day 7 p.i. **(D)** Bar graphs represent percentages of CD4^+^ T cells secreting cytokines IL-10 and IL-4 from anti-CD25 + anti-GITR treated mice or uninfected IgG control at day 7 p.i. All values represent mean ± SD values from three independent experiments with at least 3–4 animals/group. *p*-Value of ≤0.05, ≤0.01, and≤0.001 was considered significant, highly significant and very highly significant and marked with *, **, and ***, respectively.

To evaluate the effect of anti-CD25 + anti-GITR treatment on the polarization of splenic MΦs, we measured IL-4 and IL-10 levels at day 7 p.i., i.e., 24 h after the last dose of anti-CD25 + anti-GITR antibodies and found significantly reduced IL-10 (*p* ≤ 0.05), but heightened IL-4 secretion (*p* ≤ 0.05) as compared to mice that received matching IgG isotypes (Figure 5A). Moreover, anti-CD25 + anti-GITR treatment also restored the T cell proliferation capacity of splenic MΦs (*p* ≤ 0.05, Figure 5B) and increased the expression of T cell activation marker CD69 on CD4^+^ T cells (Figure 5C). Activated CD4^+^ T cells also secreted significantly lower amount of IL-10 (*p* ≤ 0.05) but more IL-4 (*p* ≤ 0.05) as compared to IgG controls (Figure 5D). These results further strengthened the notion that anti-CD25 + anti-GITR treatment checked the polarization of M2 MΦs to Mregs and strengthened the antiparasitic immune response in treated animals.

### Anti-CD25 + Anti-GITR Treatment Affects the Recruitment of Eosinophil and Bregs in the Spleens of Mice

**Figure 6:**
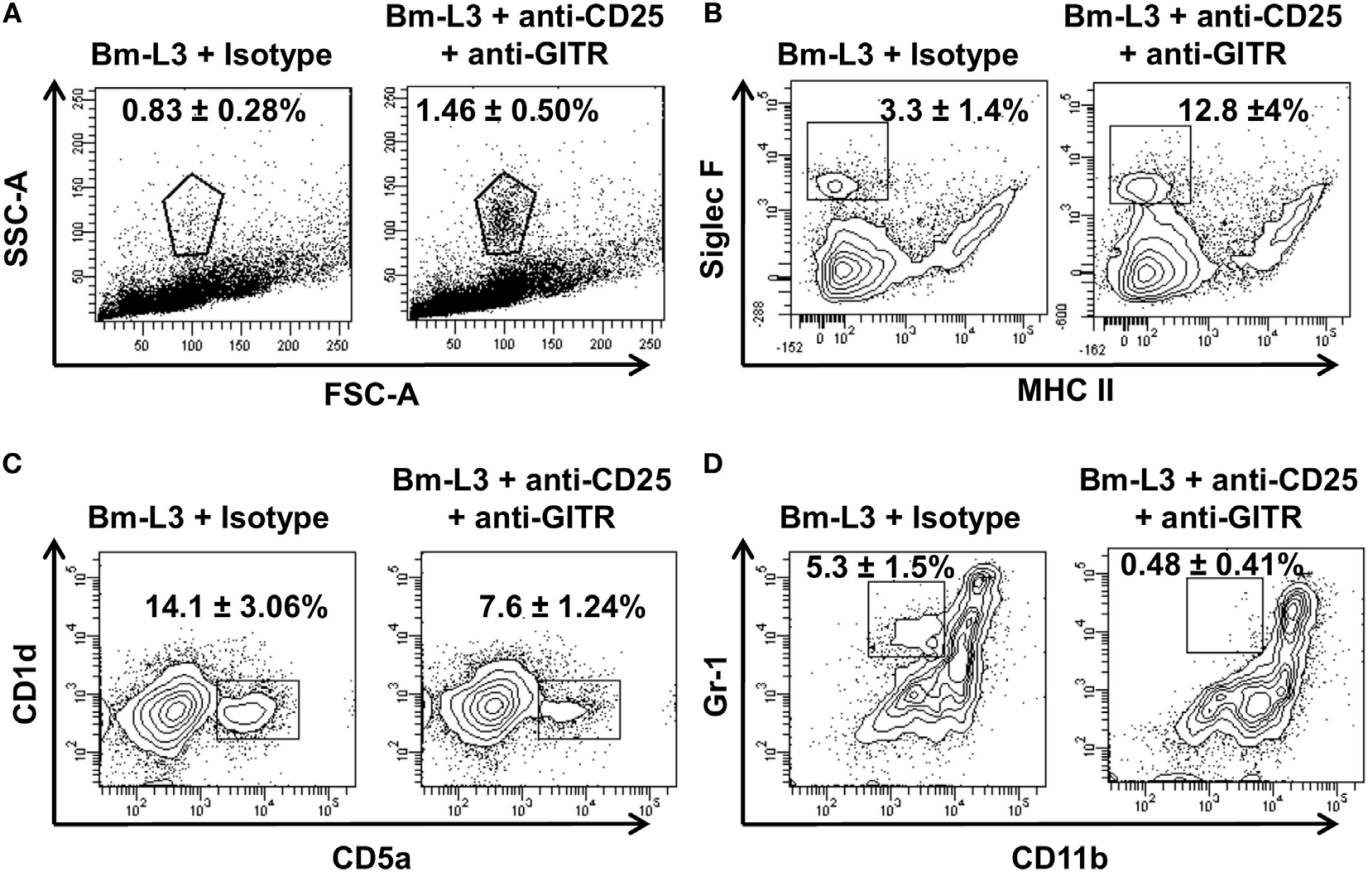
Neutralization of regulatory T-cells affects the accumulation of other leukocyte subsets. Representative flow cytometry dot plots showing **(A)** granulocytes [low forward scatter (FSC)-A, high SSC-A], **(B)** eosinophils (Sig F^+^, MHCII^−^), **(C)** regulatory B cells (CD1d^+^, CD5a^+^), and **(D)** inflammatory monocytes (Gr1^+^, CD11b^+^) present in the spleens of anti-CD25 + anti-GITR-treated mice or uninfected IgG control at day 7 p.i. All values represent mean ± SD values from three independent experiments with at least 3–4 animals/group.

To elucidate whether anti-CD25 + anti-GITR treatment had any effect on the accumulation of leukocytes, we carried out flow cytometry assisted multicolor immunophenotyping of different leukocyte populations present in the spleens of mice. We found increased percentages of granulocytes (low FSC-A, high SSC-A) in the anti-CD25 + anti-GITR-treated mice as compared to IgG controls (Figure 6A). Further confirmation revealed that most of these granulocytes were indeed eosinophils (Figure 6B, SigF^+^, MHCII^−^ cells) that were present almost four times more in anti-CD25 + anti-GITR-treated mice. However, unlike eosinophils, percentages of regulatory B cells (CD1d^+^, CD5a^hi^) and inflammatory monocytes (Gr1^+^, CD11b^+^) decreased in anti-CD25 + anti-GITR treated mice as compared to IgG controls (Figures 6C,D). These results underlined the efficacy of anti-CD25 + anti-GITR treatment in breaking immunological tolerance in mice.

### *In Vivo* Neutralization of Tregs Delays Apoptosis of M2 MΦs

**Figure 7:**
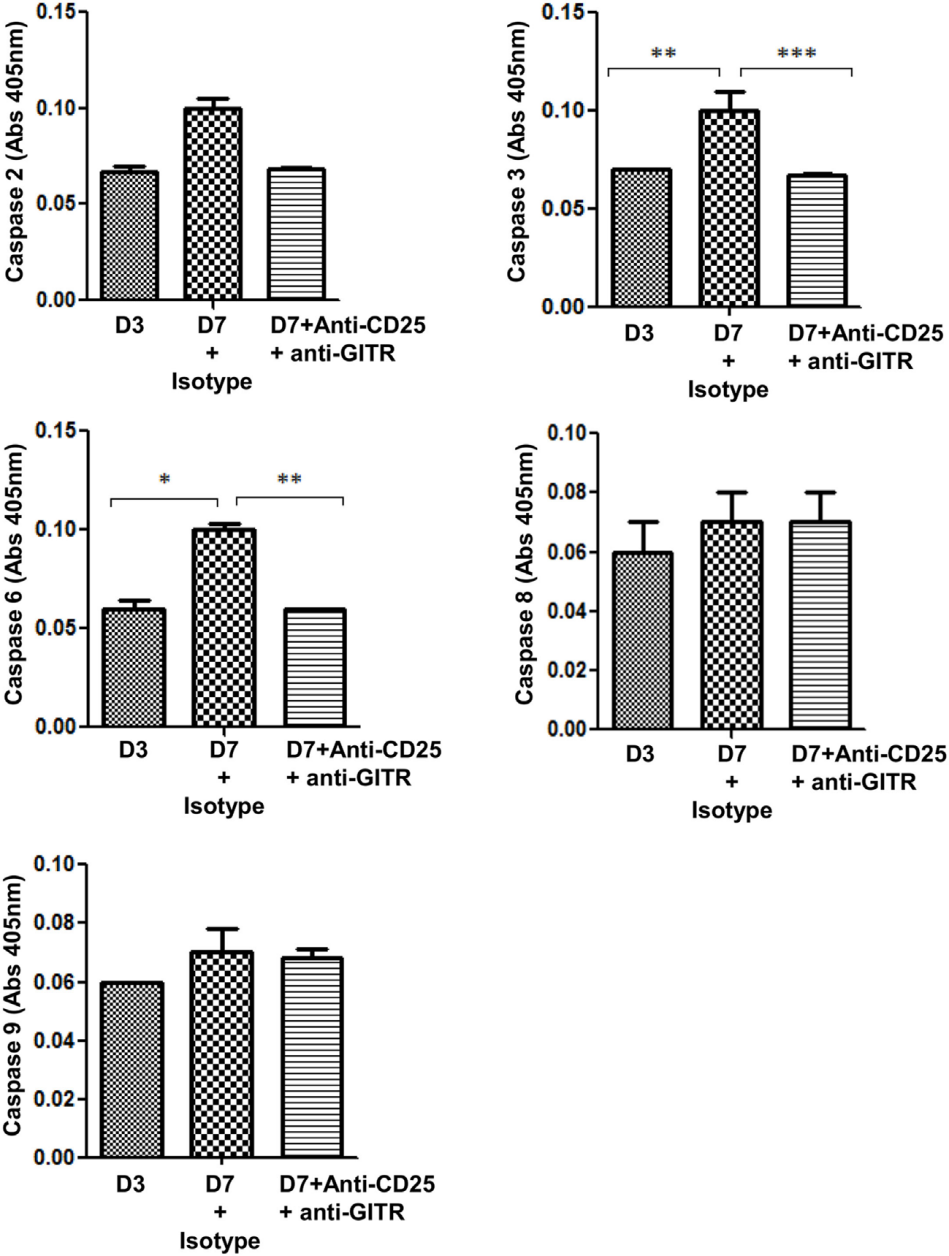
Neutralization of regulatory T-cells delays apoptosis in M2 MΦs. Bar graphs represent caspase activity in the lysate of FACS-sorted splenic MΦs from anti-CD25 + anti-GITR-treated mice or uninfected IgG control at day 7 p.i. All values represent mean ± SD values from two independent experiments with at least 3–4 animals/group. *p*-Value of ≤0.05, ≤0.01, and ≤0.001 was considered significant, highly significant, and very highly significant and marked with *, **, and ***, respectively.

Since administration of anti-CD25 + anti-GITR antibodies helped in retaining the M2 phenotype in MΦs, which contributed to reduced Bm-L3 burden, we reasoned if this treatment increased the longevity of M2 MΦs by interfering with their apoptotic machinery. We, therefore, assessed the role of caspases and found reduced expression of caspase 3 (*p* ≤ 0.001), and caspase 6 (*p* ≤ 0.01) in the cell lysate of FACS-sorted splenic MΦs from Bm-L3-infected mice that received anti-CD25 + anti-GITR treatment as compared to those that received IgG isotypes (Figure 7). Notably, no change was observed in the activity of caspase 2, 8, and 9, which not only suggested differential regulation of caspases during Bm-L3 infection, but also underlined reduced caspase activity as one of the reasons that might have prolonged survival of M2 MΦs that lead to enhanced killing of infective larvae (16).

## Discussion

Filarial parasites dampen the host immune response by interfering with the cellular and molecular mechanisms involved in the host defense, including but not limited to interfering with the functional plasticity of host MΦs (17–19). However, mechanisms that regulate the polarization of MΦs and support the establishment and survival of the parasite within the host are largely unexplored. In the present study, we infected BALB/c mice with infective larval stage 3 of the filarial parasite *Brugia malayi* and studied the polarization of host MΦs during the first week of infection. Early perturbations observed in the cytokine secreting pattern of splenic MΦs suggested alternatively activated phenotype of MΦs at day 3 post Bm-L3 infection, which gradually shifted to a regulatory phenotype by day 7 p.i. indicating that polarization of splenic MΦs was rapid and took place within the first week of infection itself. In fact, TGFβ1, an anti-inflammatory cytokine that is often listed in the same category as IL-10 was elevated at day 3 p.i., which might have aided in the generation of the M2 phenotype. Support for this notion is evident from the work done by Heisterkamp and colleagues who showed that TGFβ signaling was critical for promoting alternative macrophage activation (20).

Alternative metabolic state in murine MΦs is reflected by the nitric oxide synthase/arginase balance that is correlated with Th1/Th2 phenotype (21, 22). While Ym-1 inhibits 12/15-LOX signaling pathway that augments Th2 immunity (23–25), Fizz-1 is critically involved in wound repair, impaired worm clearance and inhibition of type 2 immunity that is suggestive of a regulatory phenotype (26). Our findings when seen in the context of Bm-L3 molting reveal an interesting scenario as Bm-L3 undergo their first molting around day 7 inside the mice and are thus highly exposed to the immune cells, so, it is in the interest of the parasite to manipulate the host immune system toward a regulatory phenotype so that no alarm signals are generated that may be harmful to the parasite. We, therefore, believe that the observed shift in the phenotype of host MΦs from M2 to Mregs around day 7 p.i. is indeed a reflection of a cleverly mastered immune evasion strategy employed by the infective larvae to escape the host immune surveillance at the earliest host–parasite interface (27, 28). In this context, it is worth considering the work done by Edwards and colleagues who demonstrated tremendous plasticity of bone marrow-derived MΦ (BMMΦ) when primed with IFN-γ, LPS, immune complexes, or IL-4 (3). Our study and that of Edwards et al. thus demonstrate the highly plastic nature of these cells, which profoundly influence the outcome of an immune response (29, 30).

Elevated levels of maturation and co-stimulatory markers and reduced support for T cell proliferation strengthened the regulatory phenotype of MΦs at day 7 p.i., which may have been due to their suppressive nature and the presence of regulatory cytokines like IL-10 at day 7 p.i. (31, 32) or even ligation of PD-L1 to its receptor PD-1 on T cells as documented in previous reports (33–35). In fact, studies have also shown that engagement of PD-L1 with its receptor PD-1 on T cells not only delivers a signal that inhibits the proliferation of T cells but also inhibits TCR-mediated IL-2 production (33–35). In addition to this, role of lipid mediators has also been documented in the suppression of T cell proliferation (36). Our observation of higher PGE2 levels corroborated with reports documenting that immune complex/Ig negatively regulate TLR4-triggered inflammatory response in MΦs through Fc gamma RIIb-dependent PGE2 production (37) which is a selective inducer of IL-12p40 (38). Not only this, induction of PGE2 by microfilariae of *Wuchereria bancrofti* and *Brugia malayi* has been documented and is believed to play a role in the host–parasite communication (39). Also, PGE2 being a potent immunomodulatory lipid mediator has also been implicated in the survival of *Trypanosoma cruzi* within the host (40). Previous reports have also documented that PGE2 enhances type 2 immunity by upregulating the production of CCL22 (41). When coalesced together, these observations provide support to the notion that elevated levels of PGE2 acted as a key regulator behind the polarization of M2 MΦs to Mregs (42).

Another notable observation of the present study was elevated expression level of TLR2 in host MΦs following Bm-L3 infection. Previous reports have not only documented the role of TLR2 in the induction of Tregs by *Schistosoma mansoni* egg antigens but activation of TLR 2 by Schistosomal lyso-phosphatidylserine has been shown to affect immune polarization also (43, 44). In addition to this, numerous reports have demonstrated interaction between TLR 2/4 to be responsible for the anti-inflammatory response induced by helminth-derived products (45).

We also observed attenuated phosphorylation of NF-κB, which is centrally involved in the regulation of inflammatory cytokines during infection and stress conditions (46, 47). Significantly upregulated levels of Erk1/2, but not that of p-p38 in M2 MΦs at day 3 p.i. suggested that MΦ polarization was mainly regulated by Erk1/2. Previous studies have demonstrated that ES-62, an excretory-secretory product of the rodent filarial nematode *Acanthocheilonema viteae* induced tyrosine phosphorylation of glycoproteins in murine macrophages (48) and modulated the activation of MAP kinases, thereby regulating cytokine production (49). In agreement with this data, our results also indicated significant upregulation of IL-4 when ERK activity was blocked, which checked the polarization of M2 MΦs to Mregs.

The heightened PTPs activity and upregulated transcript levels of six major phosphatases further supported the reasoning that the process of macrophage polarization was indeed regulated at many levels. In fact, reports have suggested that conditional removal of Shp2 in monocytes/macrophages lead to an IL-4-mediated shift toward a M2 phenotype. Additionally, an increase in arginase activity was detected in Shp2 (Δ/Δ) mice after i.p. injection of chitin, whereas Shp2-deficient MΦs showed enhanced M2 polarization and protection against schistosome egg-induced schistosomiasis (50).

Our observations of CCL22-mediated significant expansion of Tregs in the spleens of mice was corroborated with previous reports documenting the expansion of Tregs within 3–7 days of helminthes infection (51–54). As such, during chronic filarial infection in humans, Tregs secrete regulatory cytokines like CCL-4 that suppresses tumor-specific and inflammatory responses (55, 56). Also, Tregs from filariasis-infected individuals produce IL-29, a member of the IL-10 cytokine family, which exhibits antitumor and antiviral activities that programs naïve DCs to induce Treg differentiation (57, 58). Although Tregs are not a major source of IL-10 secretion during chronic human infections (59), a recent report showed that depletion of CD25^hi^ cells significantly upregulated cytokine production and the proliferation of B and T lymphocytes during patent filarial infection, which suggested that Tregs from chronically infected filarial patients were functionally more suppressive (60).

Importantly, neutralization of Tregs activity by function blocking anti-CD25 + anti-GITR antibodies not only checked the polarization of M2 MΦs to Mregs but also increased the percentages of eosinophils, which assisted in Bm-L3 killing (61). Notably, eosinophils have well-documented antihelminth properties and increased percentages of eosinophils after Treg neutralization has also been observed in cancer (62). Importantly, infection with *Strongyloides stercoralis* has been shown to induce alternatively activated macrophages within the peritoneal cavity of mice, which not only killed the worms *in vivo* but also collaborated with neutrophils and complement during the *in vitro* killing process (63). Furthermore, recent reports have also demonstrated that M2 MΦ exhibit helminth killing activity *in vivo* provided the contact time between these MΦs and the parasite is of sufficient duration, thereby implying that M2 MΦs exert a protective role during parasitic infections as their depletion also inhibit worm expulsion (5). In fact, downregulated activity of some caspases in the anti-CD25 + anti-GITR-treated mice was an interesting observation that needs to be explored in greater detail in order to elucidate the contribution of caspases in the polarization and longevity of host MΦs.

**Figure 8:**
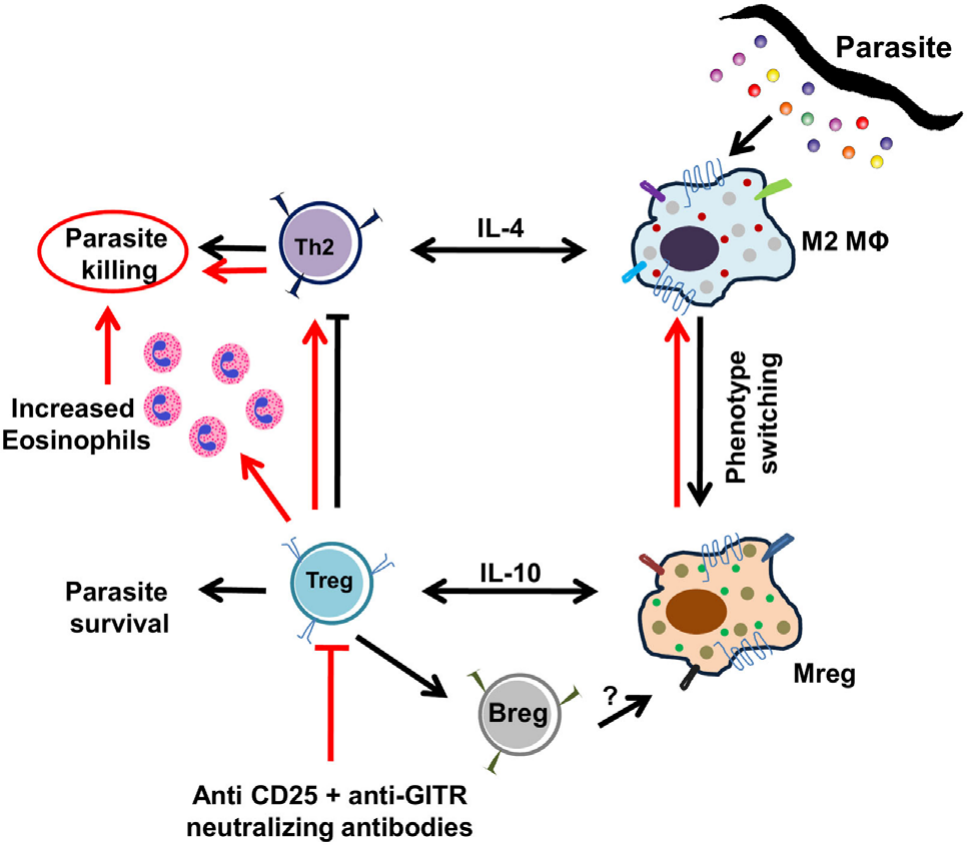
Pictorial presentation of polarization of splenic MΦs during Bm-L3 infection. Simplified view of the events associated with the polarization of splenic MΦs by infective larvae of *Brugia malayi* are shown. Black arrows represent events induced by the infective larvae to escape the host immune surveillance and establish inside the host, while red arrows show events that occur after neutralization of regulatory T cell activity.

Taken together, the present study not only documents the multifarious and intriguingly complex nature of events that regulate the polarization of host MΦs during the early phase of filarial infection (Figure 8) but also emphasizes on the hitherto unexplored cross talk between regulatory T-cells in the polarization of host MΦs which help in the establishment and survival of the infective larvae inside the host. Identification of the parasite molecules that utilize the functional plasticity of MΦs to subvert host immunity would be important for the development of effective therapeutic strategies that can halt or arrest the establishment of the filarial parasite at the earliest host–parasite interface.

## Ethics Statement

This study was carried out in accordance with the recommendations of Institutional Animal Ethics Committee. The protocol was approved by the Institutional Animal Ethics Committee.

## Author Contributions

AS and MS conceived and designed the experiments, analyzed the data, and wrote the manuscript. AS, PS, LG, and NS performed the experiments. MS and AV carried out cell sorting. SM helped in the maintenance of *Brugia malayi* infection and provided infective larvae. The authors thankfully acknowledge excellent technical support provided by O. P. Yadav for maintaining *B. malayi* infection in the laboratory.

## Conflict of Interest Statement

The authors declare that the research was conducted in the absence of any commercial or financial relationships that could be construed as a potential conflict of interest.
